# Renal biopsy findings and clinical indicators of patients with hematuria without overt proteinuria

**DOI:** 10.1007/s10157-015-1090-6

**Published:** 2015-02-13

**Authors:** Yoshie Hoshino, Toshie Kaga, Yasutomo Abe, Mariko Endo, Sachiko Wakai, Ken Tsuchiya, Kosaku Nitta

**Affiliations:** 1 Department of Medicine, Kidney Center, Tokyo Women’s Medical University, 8-1 Kawada-cho, Shinjuku-ku, Tokyo, 162-8666 Japan; 2 Department of Nephrology, Okubo Hospital Tokyo Metropolitan Health and Medical Treatment Corporation, Tokyo, Japan

**Keywords:** Hematuria without overt proteinuria, Renal biopsy, IgA nephropathy

## Abstract

**Background:**

Whether to perform a renal biopsy for isolated hematuria remains a matter of controversy. We performed renal biopsy in hematuria without overt proteinuria patients and reported the proportion of glomerulonephritis, pathological activities, and statistical analysis of indicators associated with glomerulonephritis.

**Methods:**

Among 203 patients who underwent renal biopsy in Okubo Hospital, Japan, between January 2008 and October 2013, we identified 56 patients who fulfilled the criteria: (1) urine dipstick examination shows equal to or greater than ± blood on three or more visits, (2) proteinuria <0.3 g/day (g/g Cr), (3) eGFR ≧60 ml/min/1.73 m^2^, and (4) no current medication for renal disease. We investigated biopsy findings and compared the clinical indicators in the IgA nephropathy (IgAN) and non-IgAN group.

**Results:**

The pathological diagnosis was IgAN in 35 cases (62 %), thin basement membrane disease (TBMD) in 7 (13 %), minor glomerular abnormality (MGA) in 6 (11 %), glomerular basement membrane (GBM) abnormality in 5 (9 %), and others in 3 (5 %). The histological grade of IgAN was I in 90 % and II in 10; 31 % of patients had some crescentic lesions. Comparisons between the IgAN and non-IgAN group revealed significant differences in age of onset (26 ± 13 vs. 34 ± 17 years, *p* = 0.04), serum IgA (340 ± 114 vs. 220 ± 101 mg/dl, *p* < 0.01), proteinuria (0.08 [0–0.25] vs. 0 [0–0.23] g/day [g/gCr], *p* < 0.01), and the presence of poikilocytes (40 vs. 10 %, *p* = 0.02).

**Conclusions:**

The proportion of IgAN in hematuria without overt proteinuria was high and the pathological activities were variable. Patients with hematuria without overt proteinuria should continue their medical follow-up and the best timing of biopsy may be controversial for these patients who have multiple risk factors of IgAN.

## Introduction

Isolated hematuria is a common urinary abnormal laboratory finding in clinical practice and is defined as persistent asymptomatic microscopic hematuria in the absence of hypertension, overt proteinuria, renal insufficiency, urinary tract infection, or structural abnormality of the urinary tract [1, 2]. Although isolated hematuria is sometimes an early sign of glomerulonephritis, no definite conclusions have been reached regarding the natural history of patients with isolated hematuria. Whether to perform a renal biopsy in patients with isolated hematuria remains a matter of controversy. Some nephrologists do not recommend performing a renal biopsy because diagnosis of a specific renal disease does not make any difference in terms of either patient management or treatment outcome, whereas other nephrologists recommend a renal biopsy because they think it will result in a more accurate diagnosis and prognosis [3–5]. In the present study, we performed a renal biopsy in patients with hematuria without overt proteinuria, and we report the proportion of patients with glomerulonephritis, pathological activities, and the results of statistical analysis to identify clinical indicators associated with glomerulonephritis.

## Subjects and methods

### Study population and entry criteria

Between January 2008 and October 2013, a percutaneous needle renal biopsy was performed on 203 patients in the Department of Nephrology of the Okubo Hospital Tokyo Metropolitan Health and Medical Treatment Corporation. This study was approved by the research ethics board of Okubo Hospital and conducted in accordance with the ethical standards of the Helsinki Declaration. All patients gave their informed consent to undergo the renal biopsy.

The subjects of this study were the 56 patients who fulfilled all of the following criteria: (1) urine dipstick examination shows equal to or greater than ± blood on three or more visits to our hospital, (2) maximum proteinuria between first visit and renal biopsy <0.3 g/day or g/g creatinine (Cr), (3) lowest estimated glomerular filtration rate (eGFR) ≧60 ml/min/1.73 m^2^ at the time of the renal biopsy; eGFR = 194 × serum Cr^−1.094^ × age^−0.287^ for males (× 0.739 if female) [6], and (4) no past history of medication for renal disease.

### Evaluations of the renal biopsy specimens

Each renal biopsy specimen was divided into three parts, one for light microscopy (LM), the second for immunofluorescence (IF) microscopy, and the third for electron microscopy (EM). The diagnostic criteria of the WHO classification of renal disease were used to make the pathological diagnoses [7]. An EM examination was performed when the cause of the hematuria could not be determined on the basis of the LM and IF findings. We defined mesangial IgA with C3 deposition as a “IgA nephropathy (IgAN)”, only IgA deposition without C3 composition as a “IgA deposition”, absence of abnormal LM, IF, and EM findings as a “minor glomerular abnormality (MGA)”, and showing the thinning (≦200 nm) of diffuse basement membrane as a “thin basement membrane disease (TBMD)”. Patients with no IgA lesions but some GBM abnormalities who did not fulfill the “TBMD” criteria, i.e., lysis and focal thinning, were diagnosed with a “glomerular basement membrane (GBM) abnormality”. We graded the pathological activity of IgAN based on the histological grade (H-grade) classification of the Japanese Society of Nephrology [8].

### Investigation of clinical indicators

We investigated the following clinical indicators: sex, age at the onset of hematuria (first time when the patient was told of the presence of hematuria), serum IgA level at the first visit (mg/dl), interval between the onset and renal biopsy (year), urinary erythrocyte count per high-power field (U-RBC) (/HPF), amount of proteinuria (U-prot) (g/day or g/gCr), serum Cr level at the time of renal biopsy (mg/dl), eGFR at the time of renal biopsy (ml/min/1.73 m^2^), previous gross hematuria based on medical history, hypertension (blood pressure ≧140/90 mmHg or treatment with an antihypertensive agent), urinary poikilocytes, urinary red blood cell casts, and nut-cracker phenomenon (difference between the renal vein diameter to the right and left of the superior mesenteric artery on a simple CT scan).

### Statistical analysis

Results for age at onset, serum IgA levels, and eGFR are shown as mean ± standard deviation (SD), and the results for U-RBC, U-prot, and interval from onset to biopsy are shown as median values (range). Based on the pathological diagnoses, we divided the patients into an IgAN and a non-IgAN group. Wilcoxon analysis was used on an ordinal scale and Fisher’s exact test on nominal scale to compare the groups. A *p* value <0.05 was considered to be statistically significant. A receiver operating characteristic (ROC) analysis was performed. ROC curves are graphic representations of the relationship between sensitivity and specificity of a diagnostic parameter and are drawn through potential points that represent different decision levels.

All statistical analyses were performed using JMP11 statistical package (SAS Institute Inc. Japan); *p* values <0.05 were considered to be statistically significant.

## Results

**Fig. 1:**
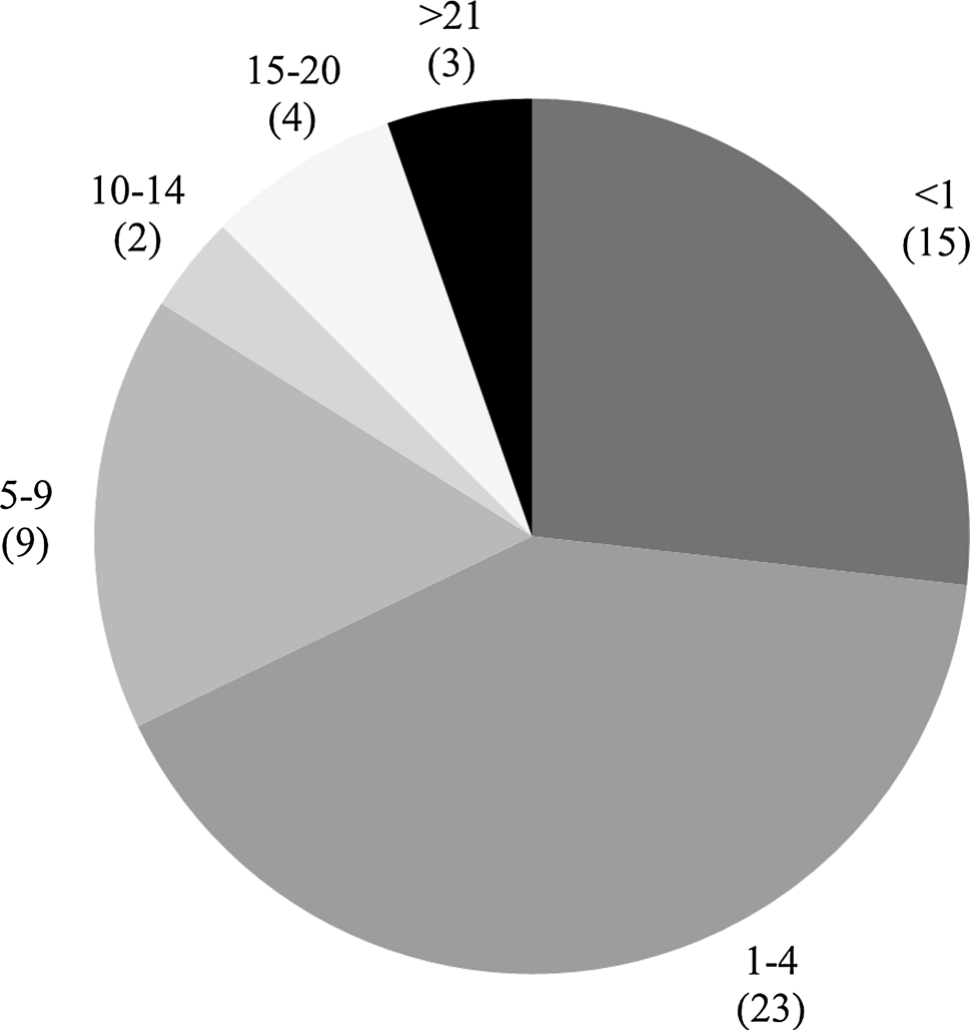
Distribution of duration to renal biopsy (years) (*n* = 56) (*n*)

The 56 subjects consisted of 32 males and 24 females, and their mean age at the time of renal biopsy was 29 ± 15 years. The distribution of duration to renal biopsy is shown in Fig. 1. A renal biopsy was performed on 3 patients to assess them as potential renal transplant donors. The mean grade of hematuria was U-RBC 10–19/HPF without overt proteinuria (>0.3 g/day or g/gCr) and renal insufficiency. The mean serum Cr and eGFR values at the time of renal biopsy were 0.82 ± 0.09 mg/dl and 92.9 ± 14.9 ml/min/1.73 m^2^ for males, and 0.62 ± 0.10 mg/dl and 91.1 ± 18.3 ml/min/1.73 m^2^ for females, respectively. Their mean serum IgA level was 295 ± 123 mg/dl, and the mean amount of U-prot was 0.04 (0–0.25) g/day (g/gCr). There were 8 patients (14 %) with U-prot ≧0.15 g/day (g/gCr). There were 18 patients (33 %) with a past history of gross hematuria, 9 (16 %) with a history of hypertension, 16 (29 %) with a history of urinary poikilocytes, and 3 (5 %) with a past history of urinary red blood cell casts. Abdominal CT was performed in 22 cases (39 %) and nut-cracker phenomenon was observed in 5 cases (Table 1).

**Table 1:** Baseline characteristics of the study population

	*n* = 56
Gender (male:female)	24:32
Age of onset (years old)^a^	29 ± 15
Serum IgA (mg/dl)^a^	295 ± 123
U-RBC (/HPF)^b^	10–19 (1–4/− >100/)
U-prot (g/day or g/gCr)^b^	0.04 (0–0.25)
Cr (mg/ml) (male:female)^a^	0.82 ± 0.09:0.62 ± 0.10
eGFR (ml/min/1.73 m^2^) (male:female)^a^	92.9 ± 14.9:91.1 ± 18.3
Duration to renal biopsy (year)^b^	2 (0–46)
Previous gross hematuria^c^	18 (33 %)
Hypertension^c^	9 (16 %)
Poikilocyte^c^	16 (29 %)
Red blood cell cast^c^	3 (5 %)
Nut-cracker phenomenon	5(IgAN3, MGA2)

Under 22 CT examination performed

*U-RBC* urinary red blood cells, *U-prot* urinary protein excretion, *Cr* creatinine, *eGFR* estimated glomerular filtration rate

^a^mean ± SD

^b^median (range)

^c^ *n* (%)

The pathological diagnosis was IgAN in 35 cases (62 %), TBMD in 7 cases (13 %), MGA in 6 cases (11 %), GBM abnormality in 5 cases (9 %), and others in 3 cases (5%; endothelial injury in 1 case and benign nephrosclerosis in 2 cases). No EM examination was performed in two cases (MGA and benign nephrosclerosis).

Glomerular lesions are compared in Table 2. Glomerulosclerosis was observed in other diseases as well as IgAN. Adhesions, crescents, and focal segmental sclerosis (FGS) lesions were observed only in IgAN. The distribution of H grade of IgAN was H-grade I in 90 % (10 of I, 2 of Ia, 13 of Ic and 8 of I a/c) and grade II (2 of II c) in the other 10 %. The glomerular lesions in IgAN consisted of adhesions in 40 %, cellular crescents in 9 %, fibrocellular crescents in 11 %, fibrous crescents in 17 %, FGS lesions in 6 %, and some form of crescentic lesions in 11 IgAN patients (31 %).

**Table 2:** Number of glomerular lesions in each disease

	Number of glomeruli^a^	Adhesion^b^	Cellular crescents	Fibrocellular crescents	Fibrous crescents	FGS lesion	Nephro-sclerosis
IgAN (35)	22 ± 11	14 (40)^b^ 10.9 ± 7^c^	3 (9)^b^ 4.3 ± 2.1^c^	4 (11)^b^ 4.8 ± 0.96^c^	6 (17)^b^ 6.7 ± 4.1^c^	2 (6)^b^ 4.5 ± 3.5^c^	18 (51)^b^ 11.8 ± 6.7^c^
TBMD (7)	16 ± 7	0	0	0	0	0	3 (43)^b^ 18 ± 14.1^c^
MGA (6)	26 ± 12	0	0	0	0	0	0
GBM abnormality (5)	14 ± 5	0	0	0	0	0	1 (20)^b^ 14^c^
Others (3)	27 ± 12	0	0	0	0	0	3 (100)^b^ 17.3 ± 16.3^c^

Rate of crescents in IgAN is 11/35 (31 %)

*IgAN* IgA nephropathy, *TBMD* thin basement membrane disease, *MGA* minor glomerular abnormality, *GBM* glomerular basement membrane

^a^ Mean ± SD

^b^ *n* (%)

^c^Rate of lesion in positive patients [lesion/total glomeruli, mean ± SD (%)]

Table 3 shows the U-RBC and U-prot levels and numbers of patients with a previous gross hematuria, complication by hypertension, presence of urinary poikilocytes, and history of urinary red blood cell casts in each disease. While U-RBC counts were almost equal among all diseases, proteinuria appeared more frequently in patients with IgAN and MGA than others. Gross hematuria was observed in most IgAN cases and in some MGA, TBMD, and GBM abnormality cases. Hypertension was observed in about 20–30 % of each disease group, but not in any of the MGA cases. Poikilocytes were observed in 14 cases of IgAN, 1 case of the GBM abnormality group, and 1 case of other disease (EM was not performed). Red blood cell casts were observed in IgAN, TBMD, and others. The nut-cracker phenomenon was observed in 3 IgAN patients.

**Table 3:** Urinary findings on each disease

	U-RBC (/HPF)^a^	U-prot (g/day or g/gCr)^a^	Previous gross hematuria^b^	Hyper-tension^b^	Poikilocyte^b^	Red blood cell cast^b^
IgAN (*n* = 35)	20 (0–100)	0.08 (0–0.3)	14 (40)	7 (20)	14 (40)	1 (3)
TBMD (*n* = 7)	5 (5–30)	0	1 (14)	1 (14)	0	1 (14)
MGA (*n* = 6)	20 (0–100)	0.01 (0–0.23)	2 (33)	0	0	0
GBM abnormality (*n* = 5)	20 (1–100)	0.00 (0–0.1)	1 (20)	1 (20)	1 (20)	0
Others (*n* = 3)	10 (1–30)	0.00 (0–0.17)	0	1 (33)^c^	1 (33)^c^	1 (33)

^a^Median (range)

^b^ *n* (%)

^c^Same person (not performed EM)

Table 4 shows the comparison of the IgAN and non-IgAN groups in terms of clinical indicators, showing significant differences in age at onset (26 ± 13 vs. 34 ± 17, *p* = 0.04), serum IgA level (340 ± 114 vs. 220 ± 101 mg/dl, *p* < 0.01), U-prot (0.08 vs. 0.00 g/day or g/gCr, *p* < 0.01), and presence of urinary poikilocytes (40 % vs. 10 %, *p* = 0.02). We performed ROC analysis to determine the cutoff point of these parameters for separating IgAN from non-IgAN. The cutoff point of age at onset was 27 years (AUC 0.66, sensitivity 0.65, specificity 0.61, positive predictive value 0.74, negative predictive value 0.52) and serum IgA level was 213 mg/dl (AUC 0.85, sensitivity 0.97, specificity 0.71, positive predictive value 0.85, negative predictive value 0.94), U-prot was 0.04 mg/dl (AUC 0.74, sensitivity 0.71, specificity 0.81, positive predictive value 0.93, negative predictive value 0.59).

**Table 4:** Comparison of clinical indicators in IgAN and non-IgAN group

	IgAN (*n* = 35)	Non-IgAN (*n* = 21)	*p*
Gender (male:female)	17:18	7:14	0.26
Age of onset (years old)^a^	26 ± 13	34 ± 17	0.04*
Serum IgA (mg/dl)^a^	340 ± 114	220 ± 101	<0.01*
U-RBC (/HPF)^b^	20–29/(1–4/− ≧100/)	10–19/(1–4/− ≧100/)	0.87
U-prot (g/day or g/gCr)^b^	0.08 (0–0.25)	0.00 (0–0.23)	<0.01*
Serum Cr (mg/ml)^a^	0.72 ± 0.15	0.68 ± 0.12	0.27
Duration to renal biopsy (year)^b^	2 (0–23)	1 (0–46)	0.42
eGFR (ml/min/1.73 m^2^)^a^	94.3 ± 18.3	87.9 ± 13.4	0.15
Previous gross hematuria^c^	14 (40)	4 (19)	0.10
Hypertension^c^	7 (20)	2 (10)	0.30
Poikilocyte^c^	14 (40)	2 (10)	0.02*
Red blood cell cast^c^	1 (3)	2 (10)	0.28

* p value <0.05 was considered statistically significant

^a^Mean ± SD

^b^Median (range)

^c^ *n* (%)

We also analyzed the prevalence rate of IgAN taking 0.15 g/day (g/gCr) of U-prot as a cutoff point, but there was no significant difference in U-prot with 0.15 g/day or more and under (20 vs. 10 %, *p* = 0.19).

## Discussion

In the present study, we made a pathological diagnosis based on the renal biopsy findings in 56 patients with hematuria without overt proteinuria, and the results showed that they had a variety of renal diseases. The most common cause was IgAN. The proportion of patients with IgAN was 62 % and with TBMD 13 %. Previous studies have reported the presence of IgAN and TBMD in 10–60 % of patients with isolated hematuria [5, 9–12]. Since the prevalence rate of IgAN and TBMD has been reported to differ according to geographic location because of differences in the frequency of urinalysis screening, indications for renal biopsy in patients with isolated hematuria, and availability of EM from country to country [13, 14], renal biopsy of patients with hematuria without overt proteinuria might yield more accurate diagnoses.

Differentiating between renal disease and urological disease is difficult without performing an invasive examination, such as renal biopsy or cystoscopy. In some patients the hematuria spontaneously resolves, but in others it is later associated with proteinuria, and it is a risk factor for end-stage renal disease [12, 14–16]. In Japan, IgAN is the most common form of glomerulonephritis as a cause of end-stage renal disease. The renal prognosis of having hematuria without overt proteinuria may be improved by early diagnosis and early treatment. In addition, especially in Japan, there are many opportunities to identify chance hematuria during school health checkups and regular checkups at work; it is meaningful to study about the timing of renal biopsy.

Clinical indicators such as younger age, high serum IgA level, proteinuria, and presence of urinary poikilocytes may be useful in differentiating IgAN from non-IgAN. Our analysis showed that the best cutoff point of age at onset was 27 years and it did not conflict with a report that pathological abnormality was the most common (69.2 %) in 20–30 years old patients with isolated hematuria [18].

The best cutoff point for IgA level was 213 mg/dl, which was lower than the clinical guidelines for IgAN in Japan [19]. It described that greater than 315 mg/dl is a frequent finding in adults with IgAN. Tomino set the cutoff point at 315 mg/dl and reported that the incidence of more than 315 mg/dl in IgAN is significantly higher than that in non-IgAN [20]. In our study, the cutoff point of 213 mg/dl has high AUC and sensitivity and negative predictive value. It shows that the possibility of IgAN is low when IgA is less than 213 mg/dl, however, it cannot be unexcludable when it is less than 315 mg/dl. There are few reports about serum IgA of IgAN and this analysis is useful.

The best cutoff point of U-prot was 0.04 mg/dl, it was also lower than expected. It may be caused by limiting population to patients with U-prot <0.3 g/day (g/g Cr) in this study. We limited the population with U-prot <0.3 g/day (g/gCr) as “patients of hematuria without overt proteinuria”, according to the “Guideline of Renal Biopsy” by Japan Society of Nephrology 2005 [21]. Recent change in guideline [22, 23] has redefined the cutoff point to ≧0.15 g/day (g/gCr) as positive for proteinuria. We also analyzed the prevalence rate of IgAN with 0.15 g/day or more and under, and there was no significant difference between two groups. It is suggested that some of the non-IgAN patients show mild proteinuria around 0.02 g/day. Based on its sensitivity and specificity, the cutoff point of U-prot 0.04 mg/dl was not a good parameter for separating IgAN from non-IgAN. And based on its high positive and low negative predictive value, the positive proteinuria is helpful for diagnosis of IgAN while the negative proteinuria cannot deny it. We should be careful while making a diagnosis based on the amount of proteinuria alone.

Poikilocytes appeared more in IgAN than in non-IgAN, but the proportion was less than 50 % in IgAN and some other diseases were also positive. Poikilocytes cannot be identified if urine is hypotonic [24] and it is identified in patients with TBMD [25], suggesting the difficulty in making a differential diagnosis based on these indicators alone.

In addition, the presence of the nut-cracker phenomenon is not useful for the exclusive diagnosis of IgAN, because there were 3 patients with nut-cracker phenomenon comorbid with IgAN. After more than 4–5 years of persistent hematuria, a renal biopsy should be performed because hematuria in persons with normal renal tissue resolves in about 4–5 years [9, 10]. However, we cannot determine the significant differences in intervals from onset to renal biopsy between the IgAN and non-IgAN groups.

The pathological activities of IgAN were variable. Some IgAN patients have minor proliferative mesangial expansion without acute and chronic lesion [H-gradeIA(−)C(−), 30 %], while others have H-gradeIIA/C (10 %) with both lesions. Glomerulosclerosis may not be a pathognomonic lesion of IgAN, because its presence has been identified in other diseases. Unexpectedly, when the total glomeruli numbers were small, a crescent as an acute lesion was identified only in IgAN and its proportion was no less than 30 % of all patients. All types of crescents including cellular, fibrocellular, and fibrous were observed. Adhesion is not determined as active lesion at H grade; it was identified in 40 % of the IgAN patients. This means that there are both old and new findings of glomerulonephritis with capillaritis without overt proteinuria and renal dysfunction, and spot urinary findings only cannot be used to estimate their activities. Therefore, it is difficult to diagnose and assess activity on the basis of glomerulonephritis according to only the one clinical indicator in patients with hematuria without overt proteinuria.

According to the present guidelines for IgAN in Japan, there is no necessity for a renal biopsy in patients with hematuria without overt proteinuria because the prognosis seems to be good. Renal biopsy has some complications, and a nephrologist should be careful in deciding to investigate by biopsy. However, Feng reported that among prognoses of pediatrics with isolated hematuria (U-prot <0.1 g/day), 6 % had adverse renal events during their follow-up period of 10 years [26]. Lee also reported that also even clinically early IgAN patients (eGFR ≧60 ml/min/1.73 m^2^, U-prot <0.5 g/day) can show a progressive disease trajectory, and 15 % progressed to ESRD during 30 years [27]. Also, Ieiri reported that the long duration of IgAN led to lower clinical remission [17].

Therefore, clinical nephrologists should carefully observe patients with hematuria without overt proteinuria and not let them drop out of their medical follow-up. They should find out the best timing for biopsy and investigation of treatment. Especially, for those patients who have multiple indicators, such as younger age, higher serum IgA level, positive poikilocytes, or some social problems such as donor and high risk of dropout, it may be useful to decide on the therapeutic principle.

The present study had several limitations. First, the sample size was small and had a selection bias for subjects. Usually, routine criteria of renal biopsy in our hospital is sustained proteinuria (+)–(2+) or 0.3–0.5 g/day (g/gCr) according to the guideline of renal biopsy [18]. With the existence of special IgAN outpatient division in our hospital, we accepted some patients who did not fulfill the criteria came and wanted to receive a thorough examination to determine the presence or absence of IgAN. In addition, among the subjects there were three candidates for renal transplant donor and had large selection bias. We obtained informed consent from all patients before the biopsy after explaining the possibility of TBMD or nut-cracker phenomenon and spontaneous remission in IgAN. Many patients desire a definitive diagnosis that enables them to decide on future plans: to pass examinations at school age and to find employment at working age. Women in the reproductive age may get married or pregnant after identifying the cause of hematuria. Nephrologists should consider the indication of renal biopsy on the basis of the patient’s social background as well as their medical data.

Second, all IgAN patients in this study received tonsillectomy and/or steroid and were led to complete. Because they all wanted to cure IgAN and they had a common reason for receiving the biopsy among them. However, it is difficult for us to observe their natural history.

As a future project, it is necessary to perform a longitudinal study to investigate the long-term outcome in these IgAN patients without overt proteinuria for 10 years or more. It may give us a new suggestion of the best timing for renal biopsy.

## Conclusions

The results of this study showed that a high proportion of the patients with hematuria without overt proteinuria had IgAN. It is difficult to diagnose glomerulonephritis or assess its activity on the basis of only one clinical indicator. It may be useful to extend indications of renal biopsy by the comprehensive consideration of medical data and social backgrounds.

## References

[1] Tomson C, Porter T (2002). Asymptomatic microscopic or dipstick hematuria in adults: which investigations for which patients? A review of the evidence. BJU Int.

[2] Tophama PS, Jethwa A, Watkins M, Rees Y, Feehally J (2004). The value of urine screening in a young adult population. Fam Pract.

[3] McGregor DO, Lynn KL, Balley RR, Robson RA, Gardner J (1998). Clinical audit of the use of renal biopsy in the management of isolated microscopic hematuria. Clin Nephrol.

[4] Fuiano G, Mazza G, Comi N, Caqlioti A, De Nicola L, Iodice C (2000). Current indications for renal biopsy: a questionnaire-based survey. Am J Kidney Dis.

[5] Shen P, He L, Jiang Y, Wang C, Chen M (2007). Useful indicators for performing renal biopsy in adult patients with isolated microscopic haematuria. Int J Clin Pract.

[6] Matsuo S, Imai E, Horio M, Yasuda Y, Tomita K, Nitta K (2009). Revised equations for estimated GFR from serum creatinine in Japan. Am J Kidney Dis.

[7] Churg J, Bernstein J, Glassock RJ (1995). WHO monograph. renal disease: classification and atlas of glomerular diseases.

[8] Matsuo S, Kawamura T, Joh K, Utsunomiya Y, Okonogi H, Miyazaki Y (2011). IgA glomerulonephritis medical-examination indicator. Nihon Jinzo Gakkai Shi.

[9] Yamagata K, Yamagata Y, Kobayashi M, Koyama A (1996). A long-term follow-up study of asymptomatic hematuria and/or proteinuria in adults. Clin Nephrol.

[10] Nieuwhof C, Doorenbos C, Grave W, de Heer F, de Leeuw P, Zeppenfeldt E, van Breda Vriesman PJ (1996). A prospective study of the natural history of idiopathic non-proteinuric hematuria. Kidney Int.

[11] Yamagata K, Takahashi H, Tomida C, Yamagata Y, Koyama A (2002). Prognosis of asymptomatic hematuria and/or proteinuria in men. High prevalence of IgA nephropathy among proteinuric patients found in mass screening. Nephron.

[12] Chow KM, Kwan BC, Li PK, Szeto CC (2004). Asymptomatic isolated microscopic haematuria:long-term follow-up. Q J Med.

[13] Schena FP (1990). A retrospective analysis of the natural history of primary IgA nephropathy worldwide. Am J Med.

[14] Tiebosch AT, Fredwrik PM, van Breda Vriesman PJ, Mooy JM, van Rie H, van de Wiel TW (1989). Thin-membrane nephropathy in adults with persistent hematuria. N Engl J Med.

[15] Iseki K, Iseki C, Ikemiya Y, Fukiyama K (1996). Risk of developing end-stage renal disease in a cohort of mass screening. Kidney Int.

[16] Szeto CC, Lai FM, To KF, Wong TY, Chow KM, Choi PC (2001). The natural history of immunoglobulin a nephropathy among patients with hematuria and minimal proteinuria. Am J Med.

[17] Ieiri N, Hotta O, Sato T, Taguma Y (2012). Significance of the duration of nephropathy for achieving clinical remission in patients with IgA nephropathy treated by tonsillectomy and steroid pulse therapy. Clin Exp Nephrol..

[18] Topham PS, Harper SJ, Fdurness PN, Harris KP, Walls J, Feehally J (1994). Glomerular disease as a cause of isolated microscopic haematuria. Quart J Med.

[19] Tomino Y, Sakai H (2003). Special study group (iga nephropathy) on progressive glomerular disease. Clinical guidelines for immunoglobulin a (Iga) nephropathy in Japan, second version. Clin Exp Nephrol..

[20] Maeda A, Gohda T, Funabiki K, Horikoshi S, Shirato I, Tomino Y (2003). Significance of serum IgA levels and serum IgA/C3 ratio in diagnostic analysis of patients with IgA nephropathy. J Clin Lab Anal.

[21] Hirakata H (2005). Indication and contraindication in renal biopsy. Nihon Jinzo Gakkai Shi..

[22] Japanese Society of Nephrology (2009). Evidence-based practice guideline for the treatment of CKD. Clin Exp Nephrol..

[23] Imai E, Nitta K, Iseki K, Fukagawa M, Yasuda Y, Yamagata K (2012). CKD clinical guideline 2012. Nihon Jinzo Gakkai. Shi..

[24] Miura H, Igarashi M, Tominaga M (2003). Evaluation of a new method for diagnosing the origin of urinary bleeding by the morphological characteristics of urinary red blood cells. Rinsho Byori..

[25] Köhler H, Wandel E, Brunck B (1991). Acanthocyturia––a characteristic marker for glomerular bleeding. Kidney Int.

[26] Feng CY, Xia YH, Wang WJ, Xia J, Fu HD, Wang X (2013). Persistent asymptomatic isolated hematuria in children: clinical and histopathological features and prognosis. World J Pediatr..

[27] Lee H, Hwang JH, Paik JH, Ryu HJ, Kim DK, Chin HJ (2014). Long-term prognosis of clinically early IgA nephropathy is not always favorable. BMC Nephrol..

